# Editorial Comment: Antioxidant enzyme profile and lipid peroxidation products in semen samples of testicular germ cell tumor patients submitted to orchiectomy

**DOI:** 10.1590/S1677-5538.IBJU.2016.0323.1

**Published:** 2017

**Authors:** Sandro C. Esteves

**Affiliations:** 1 ANDROFERT, Centro de Refêrencia para Reprodução Masculina, Campinas, SP, Brasil

In this issue of the *Int Braz J Urol*, Sposito and colleagues, in a collaborative effort between the Human Reproduction Unit of Federal University of São Paulo and the Animal Reproduction Department of University of São Paulo, provide interesting data concerning antioxidants and oxidants in the semen of testicular germ cell tumor (TGCT) patients (1).

The authors measured lipid peroxidation, a marker of oxidative stress (OS), and the levels of enzymatic antioxidants (catalase, glutathione peroxidase, and superoxide dismutase) in the semen of 26 men with TGTC (12 seminomas and 14 non-seminomas) subjected to unilateral orchiectomy. Measurements were carried out one month after surgery and before initiation of adjuvant therapy (if required). Twenty-six healthy men with semen analysis within normal ranges (WHO 2010 criteria) served as controls. Patients and controls were matched by age and ejaculatory abstinence. The testicular cancer patients had lower sperm count than controls, which is expected as spermatogenesis is reduced by half in adult men with solitary testis due to various causes, including orchiectomy for testicular cancer (2, 3). More importantly, both cancer groups had higher oxidative stress markers than controls, albeit not different between seminoma and non-seminoma. But notably, seminal antioxidant levels were similar between controls and orchiectomized TGCT patients.

Why is this study being editorialized? First, for the novelty; it is to my knowledge the first report to investigate oxidative markers and antioxidant levels in semen of orchiectomized testicular cancer patients. Second, the authors’ results add to the discussion about the optimal time for sperm cryopreservation. Third, Sposito and colleagues set the path for future research.

Reactive oxygen species (ROS) are formed during normal cellular metabolism and are involved in many physiological processes, including the activation of the immune system. Examples of ROS include superoxide anion (•O_2_−), hydrogen peroxide (H_2_O_2_), the extremely reactive hydroxyl radical (•OH), and the peroxyl radical (•HO_2_−) (4). An increase in ROS levels that exceeds their physiological threshold can induce cellular damage due to deleterious effects on proteins, lipids, and DNA. Indeed, ROS production in the male reproductive tract has become a real concern because of their potential toxic effects on sperm quality and function (4, 5). The extent of damage due to ROS depends on several factors, including intracellular and extracellular levels of ROS and extent of anti-oxidation in the environment. Abnormal spermatozoa, polymorphonuclear granulocytes or both, are primary sources of excessive ROS. The seminal plasma contains natural antioxidants (AOX), such as vitamins C and E, superoxide dismutase (SOD), glutathione peroxidase (GPx), and catalase, which counteract the adverse effects of ROS (6). An imbalance between ROS production and antioxidant defenses leads to oxidative stress (OS). Lipid peroxidation (LPO) is one end product of OS that causes oxidation of cell membranes, thus impairing its function. The other is sperm DNA fragmentation (SDF). The likely result of SDF is infertility (7), but it has been suggested that offspring generated from such defective sperm are at an increased risk of imprinting disorders and cancer (8). Oxidative stress is measured by direct and indirect methods. The direct assays measure ROS levels directly and include chemiluminescence, nitroblue tetrazolium test, cytochrome C reduction, to cite a few. Indirect methods measure the oxidized products or their effect at the molecular level and include myeloperoxidase test, redox potential, lipid peroxidation levels, total antioxidant capacity, SDF testing, among others. The assays principle, methodology, clinical utility, and drawbacks can be found elsewhere (5).

Although the literature is rich in studies examining the role of OS and antioxidants in male infertility, the study of Sposito and colleagues is the first to measure lipid peroxidation and antioxidant levels in the neat semen of TGCT patients subjected to orchiectomy (1). Before their study, LPO was investigated only in cryopreserved semen samples of testicular or non-testicular cancer patients. In frozen-thawed semen from TGCT men, LPO levels were not different than that of controls (9). The present study adds to the literature by demonstrating an oxidative imbalance among patients with TGCT, even after tumor removal.

Equally important is to discuss the clinical implications of Sposito’s et al. findings for testicular cancer patients banking their semen for fertility preservation. Foremost among all is perhaps the issue of when to freeze, before or after orchiectomy. On the one hand, some authors suggest cryopreservation is optimal before orchiectomy because sperm concentration decreases after surgery (10). On the other hand, others advocate sperm banking after orchiectomy, as a significant proportion of TGCT men have high SDF at diagnosis (11). Since cancer induces an overall inflammatory state with the release of cytokines and other products, it is possible that OS, including DNA damage, could be mitigated after orchiectomy. In the study of Sposito et al., although AOX levels were similar between TGCT patients and controls, LPO levels were higher in the former, thus indicating that the existing AOX could not fully protect sperm from the detrimental effect of ROS. Unfortunately, measurements of oxidation and AOX before orchiectomy were not available, thus precluding conclusion regarding the optimal time for cryopreservation. Notwithstanding, others have found that TGCT *per se* does not increase SDF, and suggest that sperm freezing done either before or after orchiectomy are equally valid (12, 13). In a recent study evaluating SDF rates among men with various diagnoses, we found SDF to be elevated in frozen-thawed semen of men with testicular cancer (14). Still, Spano et al. showed that SDF increases after both radiotherapy and chemotherapy, irrespective of the type of testicular cancer, an effect that persists for five years (15). Altogether, the existing evidence suggests OS is contributory to deterioration of semen quality in TGCT patients. The optimal time for freezing such specimens, before or after orchiectomy, is yet to be determined, but it should be carried out, unquestionably, before adjuvant therapy starts.

Lastly, Sposito’s et al. intriguing findings open the possibility for future research. Measurement of oxidants and AOX before and after orchiectomy could be very informative, as would be the investigation of AOX added to the freezing media, as a means to overcome any deleterious effect OS post-thawing, as previously suggested (16-18). While awaiting for these results, it seems sound to offer sperm banking both before and after orchiectomy, coupled with the determination of oxidative stress status (if available) or SDF testing, which is now commonplace (19). Based on the levels of such markers obtained at the time of cryopreservation, it may be decided later which specimen is safer to use for Assisted Reproductive Technology.

*Sandro C. Esteves, MD, PhD* *Medical and Scientific Director* *ANDROFERT, Centro de Refêrencia para Reprodução Masculina* *Av. Dr. Heitor Penteado, 1464 - Bairro Taquaral* *Campinas, SP, 13075-460, Brasil* *Fax: +55 19 3294-6992* *E-mail: s.esteves@androfert.com.br*
